# Modeling Trophic Structure and Ecosystem Functioning of the Small Fish‐Dominated Largest Lake of Bangladesh

**DOI:** 10.1002/ece3.73177

**Published:** 2026-03-14

**Authors:** Debashis Kumar Mondal, Chengjie Yin, Kangshun Zhao, Muhammad Farooq, Md. Abdul Halim, Ruilong Wang, Jun Xu

**Affiliations:** ^1^ State Key Laboratory of Breeding Biotechnology and Sustainable Aquaculture, State Key Laboratory of Lake and Watershed Science for Water Security, Institute of Hydrobiology Chinese Academy of Sciences Wuhan China; ^2^ University of Chinese Academy of Sciences Beijing China; ^3^ Bangladesh Fisheries Research Institute Mymensingh Bangladesh; ^4^ School of Environment and Surveying Engineering Suzhou University Suzhou China; ^5^ State Key Laboratory of Freshwater Ecology and Biotechnology, Institute of Hydrobiology Chinese Academy of Sciences Wuhan China; ^6^ International Institute of Aquaculture and Aquatic Sciences Universiti Putra Malaysia Port Dickson Malaysia; ^7^ Rural Development Academy Fisheries Division Gopalga Bangladesh; ^8^ School of Biology and Brewing Engineering Taishan University Tai'an China

**Keywords:** ecosystem management, Kaptai Lake, mass‐balance model, trophic structure

## Abstract

Ecosystem modeling is becoming increasingly important, as it reveals how energy flows and species interactions shape ecosystem stability, resilience, and the sustainability of fisheries. However, model‐based ecosystem studies are scarce for the Kaptai Lake (KL) of Bangladesh. Therefore, a mass‐balanced trophic model was built to illustrate the trophic structure and ecosystem features of KL. The model outputs indicated that the apex predator, Catfish (TL‐3.364), occupied the top trophic niches, while the overabundant (B: 3.264 t/km^2^) Clupeid (TL‐2.56) dominated the lower trophic level in the food web. The higher values of ecotrophic efficiency (> 0.5) for most of the groups indicate heavy fishing pressure. The ecosystem is Phytoplankton‐based, where two core food chains that make up the majority of the food web are the Detritus (35.12%) and primary production (64.88%). The mixed trophic impact plot revealed a substantial positive effect of Detritus and Phytoplankton on the majority of the fish groups. Clupeids displayed a negative effect on most of the fish groups, indicating a lack of major predators of this group. In the KL ecosystem, Catfish and Phytoplankton were the key predator and producer groups, respectively. The network analysis indicates that the KL is a developing ecosystem with moderate system maturity, as evidenced by total primary production and respiration ratio (2.034), total primary production and biomass ratio (71.764), and Finn Cycling Index (5.627%). The low connectance index (0.426) indicates that the food web is still linear rather than a web‐like structure and thereby vulnerable to external influences. Besides, the ever‐increasing trend of Clupeid and declination of large fish populations indicates that the KL ecosystem may encounter severe consequences in the future. Consequently, ecosystem‐based management interventions have been proposed that will help to restore food web integrity, recover vulnerable species and ensure long‐term sustainability of fisheries in this lake.

## Graphical abstract

Kaptai Lake is a developing ecosystem with a linear food web and thereby vulnerable to external influences. The apex predator, Catfish (TL‐3.364), occupied the top trophic niches, while the overabundant (B: 3.264 t/km^2^) Clupeid (TL‐2.56) dominated the lower trophic level in the food web. The higher values of ecotrophic efficiency for most of the groups indicate heavy fishing pressure.

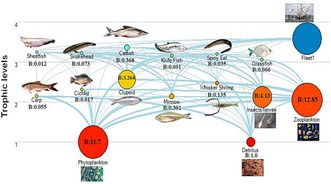

## 1 Introduction

Multiple anthropogenic stressors, including eutrophication, hydrological regulation, biological invasions, and overexploitation, may have synergetic effects on lake ecosystems. It may lead to drastic deterioration of food web patterns (Yang and Lu 2014) and show nonlinear behavior when particular limits are surpassed (Casini et al. 2009). This process has negatively impacted many lake ecosystems by reducing biodiversity, changing energy flow, and influencing nutrients recycling (Kong et al. 2016). For example, overfishing also undermines the processes and structure that keep the food web stable (Rooney et al. 2006). This may lead to catastrophic consequences, including “fishing down of the food web” (Pauly et al. 1998), collapses of food webs (Downing et al. 2012), and eventually severe regime changes (Casini et al. 2009).

Fish populations are a key component of aquatic ecosystems, but they have historically been investigated as single species (Ullah et al. 2012). Over the past few years, there is a growing recognition that conventional fisheries management approaches are inadequate and somewhat ineffective (Mohamed et al. 2005). Consequently, ecosystem‐based fisheries management has become increasingly popular as a strategy for achieving viable fisheries and maintaining healthy ecosystems (Ullah et al. 2012). In contrast to other ecosystem modeling approaches, the ‘Ecopath with Ecosim’ software is currently a popular tool for ecosystem‐based fisheries management to measure ecosystem features and show trophic linkages due to its ease of use (Villasante et al. 2016). This method was one of the first publicly available and easily accessible ecosystem‐level simulation models, which led to its widespread use as a key instrument for an ecosystem‐based method to address fisheries and associated aquatic ecosystems (Guo et al. 2018). Moreover, EwE has become a dynamic, spatial platform for ecosystem‐based management, now integrating environmental drivers for climate change analysis and advanced Management Strategy Evaluation (MSE). It has also been open‐sourced and offers scriptable access via R, greatly enhancing its flexibility and power for policy testing (Coll et al. 2009).

Kaptai Lake (KL) is one of the largest man‐made freshwater lakes in Southeast Asia (Rahman et al. 2024), historically serving the nation in different ways, including fishing and aquaculture, navigation, flood and drought resistance, biodiversity conservation, tourism, and irrigation (Suman et al. 2021). The total fish production of KL in 2022–23 is 17,056 MT, which is 0.35% of the country's total production (DoF 2023). Despite its immense importance, knowledge of overall ecosystem health is still insufficient. Furthermore, the majority of existing studies were only focused on fisheries biodiversity (Lima et al. 2023; Ahmed et al. 2003a; Suman et al. 2021), fish population dynamics and stock assessment (Rahman et al. 2024; Mondal et al. 2024; Bashar et al. 2021; Khatun et al. 2019; Khatun et al. 2023), fishing gear (Rahman et al. 2024; Hoque et al. 2014), water quality (Karmakar et al. 2011; Das et al. 2024), and fisheries management (Ahmed et al. 2006). Due to the intricate connections among species in an ecosystem, the current understanding of the trophic structure of the KL ecosystem is still poor, with the exception of a preliminary study on this lake (Khatun et al. 2021). However, that study overlooked the temporal variation of species composition and mean trophic level of catch (MTLc). Moreover, that study is insufficient for scientifically managing and understanding the lake resources under the scenario of gradual increase of small fishes and decrease of carps and apex predators in recent decades. An ecosystem‐based analysis usually requires a large amount of basic ecological data, making it a data‐intensive field of study (Guo et al. 2018). That's why, till now, no extensive ecosystem‐based study is available on the changes in ecosystem attributes and food web structure addressing the changes of MTLc over a long period and massive shifts in species composition, especially Carps and Clupeids, over the last couple of decades. Therefore, a holistic ecological investigation is fundamental for addressing these issues and assessing potential ecological consequences of anthropogenic and natural influences in response to current demand.

In recent years, some threats to the lake ecosystem and food web have emerged, including overexploitation, destruction of natural breeding grounds of Carps due to siltation, a dramatic increase of small fish, water quality deterioration, introduction of exotic species, and illegal fishing threatening the viability of the lake's fisheries (Suman et al. 2021; Khatun et al. 2023). Recent studies showed that Carp production declined from 81% to 1.56%, whereas two small fishes under the Clupeid family ( *Gudusia chapra* and *Corica soborna* ) jumped from 3% to 64% of the total production from 1965 to 2018 (Suman et al. 2021; Khatun et al. 2023). This dominance significantly influences the biomass and shape of the planktonic ecosystem for food and niche, which can induce additional trophic cascading impacts on other planktivore fishes and apex predatory species, particularly Carps. Therefore, it is crucial to make a comprehensive study to assess their roles and the shape and functioning of lake ecosystem to develop an effective strategy for maintaining aquatic ecosystem health.

The objectives of the present study were (1) to develop an Ecopath mass‐balance model for KL and to assess the trophic structure, ecosystem health, and the roles and functions of target species (e.g., Catfish and Clupeid) in KL and (2) to identify the primary drivers and underlying mechanisms affecting food web structure and ecosystem functioning of KL, and to provide management experience reference for lakes with similar ecological environments and management conditions.

## 2 Materials and Methods

### 2.1 Study Area

**FIGURE 1:**
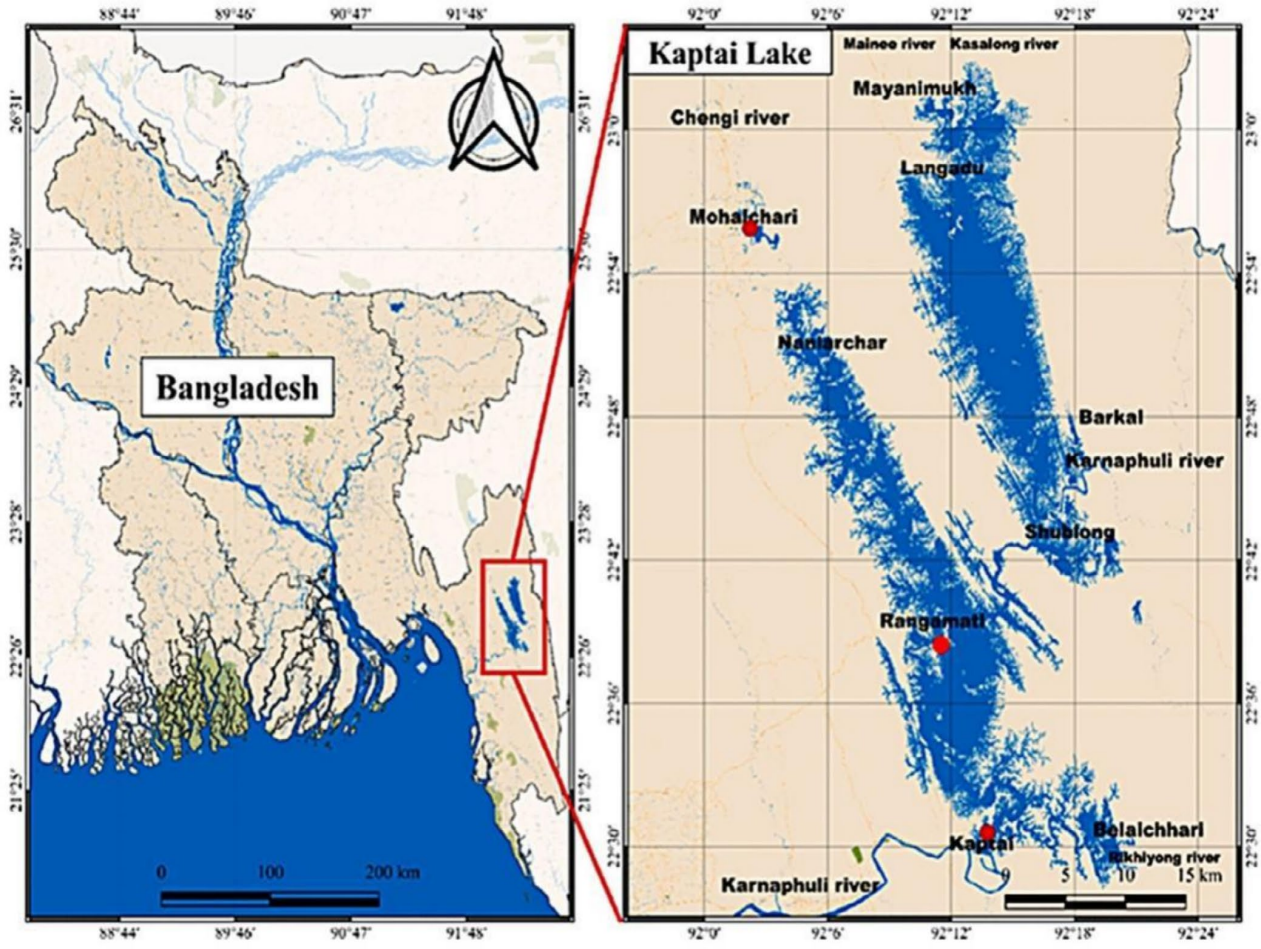
Geographical location of Kaptai Lake. Red circle indicate the study area (Khatun et al. 2021).

The current study was carried out in the KL (22°22′–23°18′ N; 92°00′–92°26′ E) of the Chittagong hill tract region of Bangladesh (Figure 1). The total area of this lake is 68,800 ha including a water surface area of 58,300 ha and the average depth is 9 m (Lima et al. 2023). The mean temperature of the KL ecosystem is 27°C. It is connected with the nutrient‐rich discharge of the Karnafuli River. However, siltation has reduced 25% of the lake volume over the last 30 years and destroyed four adjacent natural breeding grounds of Carps (Suman et al. 2021; Lima et al. 2023). Fisheries play a vital role in the economy of the Chittagong Hill Tracts region, and Kaptai Lake is at the center of this industry. The lake supports thousands of fishermen who rely on it for their livelihoods. Fish from Kaptai Lake is supplied to local and national markets, with some species being exported to international destinations. The lake's fishery also supports ancillary industries such as boat‐building, fish processing, and gear manufacturing.

### 2.2 Ecopath Modeling Approach

A mass‐balanced ecosystem model for KL was designed employing the Ecopath with Ecosim (EwE) software (version 6.6.7). This model designates all living organisms as a set of interconnected functional groups. These groups are assumed to be mass balanced within a somewhat stable ecosystem, as illustrated with the following formula:

(1) $$B_{i}.{\left(\frac{P}{B}\right)}_{i}\cdot {\mathrm{EE}}_{i}-\sum_{j=1}^{i}B_{j}\cdot {\left(\frac{Q}{B}\right)}_{j}\cdot {\mathrm{DC}}_{\mathrm{ji}}-{\mathrm{EX}}_{i}=0$$

where *B*_*i*_ represents the biomass of prey group *i*, (*P*/*B*)_*i*_ denotes production/biomass ratio of that group, and EE_*i*_ is the ecotrophic efficiency. *B*_*j*_ indicates the biomass of predator group *j*, (*Q*/*B*)_*j*_ represents food export or consumption per unit biomass of *j*, DC_*ji*_ implies the proportion of prey *i* in the diet of *j*, and EX_*i*_ denotes export of *i*. DC and EX are mandatory to enter always, whereas entry of any of the other four parameters (*B*, *P*/*B*, *Q*/*B*, and EE) is optional.

### 2.3 Model Construction

#### 2.3.1 Fishery Landing Data

Fishery catch data of KL were collected from the 3 landing centers (Figure 1) of Bangladesh Fisheries Development Corporation (BFDC) adjacent to this lake in the year of 2024 (Table 1). Fishermen bring their catches to these landing centers due to excellent infrastructure, communication, and icing facilities (Suman et al. 2021).

**TABLE 1:** Life history parameters of the representative species of different functional groups in Kaptai Lake along with their annual catch.

Species	Annual Catch (t)	L∞ (cm)	K (/yr)	a	b	W∞ (g)	M (/yr)	F (/yr)	Z (/yr)	E	References
Sheatfish	**7.599**										
*Wallago attu*	6.667	99.75	1.3	0.0074	3.05	9259	1.47	1.9	3.37	0.56	Thella et al. (2018)
*Ompok pabda*	0.932	21	1	0.0048	3.1	60.3	1.92	0.29	2.22	0.13	Gupta et al. (2015)
Snakehead	**22.961**										
*Channa punctata*	1.237	24	0.9	0.0457	2.83	368	2.16	1.57	3.13	0.5	Mustafa and De Graaf (2008)
*Channa striata*	6.389	63	0.77	0.0107	2.933	2027	1.24	1.27	2.51	0.51	Shikha Datta et al. (2023)
*Channa marulius*	15.335	40.95	0.3	0.006	3.033	467	0.68	0.5	1.18	0.42	Guilin et al. (2019)
Catfish	**350.275**										
*Sperata seenghala*	225.055	117.6	0.37	0.004	3.047	8139	0.56	0.44	1	0.44	Memon et al. (2017)
*Ailia coila*	57.822	18.9	0.68	0.08	3.13	791	0.81	1.84	2.65	0.69	Ray et al. (2022)
*Eutropiichthys vacha*	6.653	44.4	0.7	0.0088	2.96	662	1.27	2.96	4.23	0.7	Bashar et al. (2021)
*Mystus tengara*	51.331	11.19	0.72	0.0125	3.01	18	1.72	1.28	3	0.43	Islam et al. (2024)
*Mystus cavasius*	3.261	21.5	0.56	0.0166	3.03	180.9	1.35	2.51	3.86	0.5	Thippeswamy et al. (2022)
*Heteropneustes fossilis*	6.153	25	0.5	0.0012	3.5	93.7	1.15	0.8	1.95	0.41	Mustafa and De Graaf (2008)
Knifefish	**18.014**										
*Notopterus notopterus*	14.543	34.91	0.38	0.0176	2.99	722	0.91	0.28	1.19	0.24	Mustafa et al. (2014)
*Notopterus chitala*	3.471	82.95	0.39	0.0126	2.896	4550	0.64	0.4	1.04	0.38	Memon et al. (2022)
Spiny eel	**8.881**										
*Mastacembelus armatus*	8.881	58.8	0.13	0.004	2.8733	485	0.32	0.43	0.75	0.57	Bin et al. (2019)
Glassfish	**57.13**										
*Chanda nama*	57.13	10.25	0.55	0.0071	3.13	10.5	1.63	1.48	3.11	0.48	Asadujjaman et al. (2023)
Carp	**41.345**										
*Labeo rohita*	6.538	93.28	0.92	0.0154	2.971	10,966	1.22	1.31	2.53	0.5	Ahmed et al. (2005)
*Catla catla*	8.917	93.68	0.57	0.0047	3.293	14,623	0.9	1.01	1.91	0.53	Ahmed et al. (2003a)
*Cirrhinus cirrhosus*	1.751	85.07	0.43	0.0114	3.001	7057	0.75	1.29	2.04	0.63	Ahmed et al. (2004)
*Labeo calbasu*	6.655	52.5	0.76	0.0036	3.47	3352	1.25	1.64	2.89	0.56	Haroon et al. (2002)
*Labeo bata*	17.484	40.2	1.3	0.012	3.08	1048	0.96	1.13	2.09	0.54	Dwivedi (2013)
Cichlid	**6.076**										
*Oreochromis niloticus*	6.076	55.59	0.39	0.0366	2.844	3361	0.8	0.59	1.39	0.42	Ahmed et al. (2003b)
Clupeid	**5337.94**										
*Corica soborna*	2291.67	5.7	2.62	0.0084	2.968	1.5	5.11	3.4	8.51	0.4	FishBase
*Gudusia chapra*	3046.27	19.95	0.89	0.0079	2.98	59.5	1.85	2.21	4.06	0.54	Mondal et al. (2024)
Minnow	**359.234**										
*Amblypharyngodon mola*	359.234	10.47	0.95	0.0067	3.21	12.7	1.22	2.04	3.26	0.63	Azadi and Mamun (2009)

*Note:* In the Species column, a bold value indicates the group name of fish, under which several fish species belonging to the respective group are listed.In the Annual Catch column, a bold value indicates the cumulative total catch of all mentioned species belonging to the respective group.

Abbreviations: E, exploitation rate; F, fishing mortality; K, growth coefficient; L∞, asymptotic length; M, natural mortality; Z, total mortality.

#### 2.3.2 Functional Groups

This study incorporated fifteen functional groups, comprising ten fish groups with twenty‐four fish species and five non‐fish groups, including prawn, Phytoplankton, Zooplankton, Insects/larvae, and Detritus. Species were selected considering their relative abundance in total landings and their role in the whole ecosystem. The Zooplankton groups include Copepods, cladocerans, Crustacea, Protozoa and rotifers. The Phytoplankton group comprises Chlorophyceae, Cyanophyceae, Euglenophyceae and Dinophyceae. Aquatic insects and larvae are crucial food for some Carps which include caddisflies, midges, mayflies, insect larvae, caddisworms, gastropods, oligochaetes and bivalves (Khatun et al. 2021; Lima et al. 2023). Macrophytes provide a feeding habitat for specific Carps and Minnows; however, their low dietary presence and minimal ecotrophic efficiency (0.008) in a similar reservoir ecosystem in the same geographical area led to their exclusion from the current study (Khatun et al. 2021; Panikkar et al. 2015). The functional groups were classified based on their biological and ecological connections, including food and feeding behaviors, spatial distributions, life history parameters, ecological roles and so on (Tesfaye and Wolff 2018). For each functional group, data on diet composition, population parameters (Table 1), and other information were taken preferably from the literatures of same ecosystem. If any information was lacking, data from similar ecosystems (Lake) in the same geographical area and FishBase (www.fishbase.org; Froese and Pauly 2025) were utilized to fill up the gaps and to make the model more authentic.

#### 2.3.3 Model Parameters

##### 2.3.3.1 Biomass (B)

Biomass (t/km^2^) of ecological groups was calculated using the following equation (Gulland 1971):

(2) $$B=\frac{Y}{F\;},$$

where ‘*Y*’ represents the annual catch of the respective group and ‘*F*’ indicates the coefficient of fishing mortality. The total amount of biomass of most of the fish groups was calculated using the previous life history data derived from the field surveys and stock assessments of this lake. Due to the lack of dependable biomass data and heavy exploitation rate for Snakehead, the EE value was set at 0.95 to compute the biomass data of this group in the EwE model (Table 2). Biomass of Whisker Shrimp, Insects/larvae, Zooplankton and Phytoplankton was taken from the field survey of previous studies in the same ecosystem (Lima et al. 2023; Khatun et al. 2021). The biomass of Detritus was calculated using an experimental equation derived from primary production along with the euphotic depth of KL, as proposed by Christensen et al. (2008):

(3) logD=0.954logPP+0.863logE−2.41

where ‘*D*’ represents the biomass of Detritus in g·C/m^2^, ‘PP’ indicates primary production in g·C/m^2^, and ‘*E*’ signifies the euphotic depth in meter. The depth of the euphotic region was determined in the following way: *E* = 2.5 × SD (Secchi depth in meter). The average ‘SD' of KL was 1.83 m. So, *E* = 4.575 m.

**TABLE 2:** Input values and computed parameters (bold face) of Kaptai Lake after mass‐balancing.

Functional groups	TL	B (t/km^2^)	P/B (/yr)	Q/B (/yr)	EE (/yr)	P/Q (/yr)	FD (t/km^2^/yr)	NE	OI	P/R	R/A
1. Sheatfish	**3.243**	0.012	2.8	10.9	**0.551**	**0.257**	**0.041**	**0.321**	**0.508**	**0.473**	**0.679**
2. Snakehead	**3.222**	**0.073**	2.27	8.93	0.95	**0.254**	**0.138**	**0.318**	**0.236**	**0.466**	**0.682**
3. Catfish	**3.364**	0.368	2.78	12.12	**0.701**	**0.229**	**1.2**	**0.287**	**0.244**	**0.402**	**0.713**
4. Knifefish	**3.152**	0.091	1.12	7.4	**0.418**	**0.151**	**0.194**	**0.189**	**0.248**	**0.233**	**0.811**
5. Spiny eel	**3.202**	0.035	0.75	9.5	**0.768**	**0.079**	**0.073**	**0.099**	**0.268**	**0.109**	**0.901**
6. Glassfish	**3.054**	0.066	3.11	23.3	**0.936**	**0.133**	**0.322**	**0.167**	**0.108**	**0.200**	**0.833**
7. Carp	**2.143**	0.055	2.29	19.14	**0.971**	**0.12**	**0.216**	**0.149**	**0.136**	**0.176**	**0.850**
8. Cichlid	**2.315**	0.017	1.39	14.1	**0.873**	**0.098**	**0.053**	**0.123**	**0.237**	**0.140**	**0.877**
9. Clupeid	**2.560**	3.264	6.29	35.3	**0.612**	**0.178**	**31.014**	**0.222**	**0.285**	**0.286**	**0.777**
10. Minnow	**2.137**	0.302	3.26	76.2	**0.988**	**0.043**	**4.615**	**0.053**	**0.125**	**0.056**	**0.946**
11. Whisker Shrimp	**2.471**	0.135	3.16	12.64	**0.982**	**0.25**	**0.350**	**0.312**	**0.299**	**0.454**	**0.687**
12. Insects/larvae	**2.131**	4.125	4	30	**0.833**	**0.133**	**27.506**	**0.167**	**0.121**	**0.2**	**0.833**
13. Zooplankton	**2.053**	12.85	35	140	**0.353**	**0.25**	**650.748**	**0.312**	**0.053**	**0.454**	**0.687**
14. Phytoplankton	**1**	11.7	203	—	**0.649**	—	**834.103**	—	—	—	—
15. Detritus	**1**	1	—	—	**0.228**	—	—	—	**0.298**	—	—

*Note:* Bold values represent computations performed by the EwE software, as indicated in the table's title.

Abbreviations: B, biomass; EE, ecotrophic efficiency; FD, flow to detritus; NE, net efficiency; OI, omnivory index; P/B, production rate; P/Q, production‐consumption ratio; P/R, production‐respiration ratio; Q/B, consumption rate; R/A, respiration‐assimilation ratio; TL, trophic Level.

##### 2.3.3.2 Production/Biomass (P/B)

The Production and Biomass ratio (P/B) of fish groups was calculated from the empirical equation of Beverton and Holt (2012) as follows (Christensen et al. 2005):

(4) $$Z=M+F=\frac{P}{B}=K\;\frac{L_{\infty }-L_{\mathrm{avg}}}{L_{\mathrm{avg}}-L_{c}}$$

where ‘*Z*’, ‘*M*’ and ‘*F*’ represent the total mortality (year^−1^), natural mortality (year^−1^) and fishing mortality (year^−1^) respectively. *K*, *L*_∞_, *L*_avg_ and *L*_*c*_ define the Von Bertalanffy Growth Function (VBGF) (year‐1), asymptotic length of fish (cm), average length (cm) of fishes in the population, and mean length at first capture (cm), respectively. The P/B (*Z*) values of fish groups were taken preferably from the existing literature of the same ecosystem or FishBase. The values of P/B for Whisker Shrimp, insect/larvae, Zooplankton and Phytoplankton were used from the previous study of the same ecosystem (Khatun et al. 2021) (Table 1).

##### 2.3.3.3 Relative Food Consumption (Q/B)

Consumption/Biomass (Q/B) ratio of each fish group was estimated through the following empirical equation suggested by Palomares and Pauly (1998):

(5) $$\mathrm{Log}\;\left(Q/B\right)=7.964-0.204\;\log\;W\infty -1.96\text{5T}+0.08\text{3A}+0.53\text{2h}+0.39\text{8d},$$

where ‘W∞’ is the asymptotic weight (g) derived from the VBGF parameter ‘L∞’ and the length‐weight relationship of the respective species (Table 1). In the current study, ‘*a*’ and ‘*b*’ values of the length‐weight relationship for all fish species were taken preferably from previous articles of the same ecosystem and FishBase to estimate ‘W∞’. ‘T' is the average annual temperature of the respective ecosystem, which is expressed as 1000/(Tc + 273.1). Here ‘Tc’ denotes the annual mean temperature of water surface (27°C) (Khatun et al. 2021). ‘*A*’ is the aspect ratio of the caudal fin, which indicates metabolic activity, and was obtained from the equation *A = h* ^2^/s (Sambilay Jr. 1990), where ‘h’ is the measured height and ‘s’ is the surface area of the caudal fin of fishes; ‘*h*’ and ‘*d*’ are dummy variables that represent feeding type. For carnivore, *h* = 0, *d* = 0; for detrivores, *h* = 0, *d* = 1; and for herbivores, *h* = 1, *d* = 0.

To estimate Q/B of fish, morphometric data of at least 10 adult individuals from each species were collected from field sampling. For the group of Whisker Shrimp, insect/larvae and Zooplankton, Q/B was taken from the previous study of the same ecosystem (Khatun et al. 2021) (Table 2).

#### 2.3.4 Diet Composition

The dietary data for different fish groups were primarily obtained from the prior dietary studies of the same lake, while the remaining data were sourced from previous studies on similar ecosystems (lakes) within the same geographical region to create a diet matrix (Table 3) which supported building a more authentic model on this lake. The data sources of diet along with references are shown in Table S1 of the supplementary file.

**TABLE 3:** Diet composition of the functional groups in the Ecopath model of the Kaptai Lake.

Prey/predator	1	2	3	4	5	6	7	8	9	10	11	12	13
Sheatfish	0	0.002	0.001	0	0	0	0	0	0	0	0	0	0
Snakehead	0	0	0.026	0	0	0	0	0	0	0	0	0	0
Catfish	0.093	0.024	0.020	0	0	0	0	0	0	0	0	0	0
Knifefish	0	0	0.003	0	0	0	0	0	0	0	0	0	0
Spiny eel	0	0.005	0.0003	0	0	0	0	0	0	0	0	0	0
Glassfish	0.093	0.026	0.002	0.037	0.093	0	0	0	0	0	0	0	0
Carp	0.072	0	0.01	0	0	0	0	0	0	0	0	0	0
Cichlid	0.031	0	0.002	0	0	0	0	0	0	0	0	0	0
Clupeid	0.155	0.243	0.640	0.309	0.229	0.055	0	0	0	0	0	0	0
Minnow	0.186	0.213	0.013	0.1	0.206	0	0	0	0	0	0	0	0
Whisker Shrimp	0.175	0.1	0.005	0.013	0.05	0.035	0	0	0	0	0.011	0	0
Insects/larvae	0	0.196	0.131	0.278	0.286	0.504	0.075	0.05	0.1	0	0.208	0	0
Zooplankton	0	0.097	0.061	0.139	0.023	0.329	0.055	0.246	0.425	0.13	0.208	0.125	0.05
Phytoplankton	0.082	0.041	0.044	0.077	0.069	0.044	0.52	0.7	0.375	0.85	0.104	0.305	0.8
Detritus	0.113	0.052	0.044	0.046	0.046	0.033	0.35	0.004	0.1	0.02	0.469	0.57	0.15
Import	0	0	0	0	0	0	0	0	0	0	0	0	0
Sum	1	1	1	1	1	1	1	1	1	1	1	1	1

#### 2.3.5 Ecotrophic Efficiency (EE)

Ecotrophic efficiency (EE) is the fraction of production of a group that is utilized within the system or harvested by fishery. The EE value ranges between 0 and 1. If any EE value approaches 1 for a group, it indicates that the group is vulnerable to fishing or predation pressure (Christensen et al. 2005). Since there is no empirical equation for estimating EE, it is computed by the other parameters in the model (Christensen et al. 2000).

### 2.4 Balancing of Model

Each group must achieve mass‐balance in accordance with Ecopath equation‐1, which means that exports, biomass, catches, and consumption cannot surpass production. So, model balancing requires the modification of input parameters to ensure that EE remains below 1 (Harvey et al. 2012). Under certain conditions, the EE value of a functional group may exceed one, signifying that the demand for the respective functional group is extremely high to be sustainable. A high EE value implies that the “other mortality” of that functional group is very low, but fishing mortality or predation mortality is elevated (Christensen et al. 2008). For mass balancing of the model, we slightly adjusted the diet matrix manually (Christensen et al. 2005), as the feeding habits of some species are highly variable, relying primarily on food sources available in their habitat and a lack of reliable data in different literatures. To balance the model, the major predation relationship responsible for the unbalanced groups in the model output was identified. Subsequently, we reduced the amount of imbalanced groups in the diet of predators as follows (Heymans et al. 2016; Wang et al. 2022):

(6) $$C_{\mathrm{ij}}\left[\frac{\left({\mathrm{DC}}_{\mathrm{ij}\;\max}-{\bar{\mathrm{DC}}}_{\mathrm{ij}}\right)}{\sum_{j=1\to n}\left({\mathrm{DC}}_{\mathrm{ij}\;\max}-{\bar{\mathrm{DC}}}_{\mathrm{ij}}\right)}\right]\times C_{\mathrm{ik}}$$

where *i* indicate the predator that needs to modify in diet; *j* is the prey to raise in proportion, *n* is the number, and *C*_*ij*_ is the amount; *k* is the prey to reduce in proportion, and the amount is *C*_*ik*_; DC_*ij* max_ is the maximum proportion of *i* that feeds on *j*; DC_ij_ is the average proportion of *i* that feeds on *j*. After repeated diet adjustment, the EE values of all functional groups were below 1 and the model attained mass balance. The computed trophic levels of different species were cross‐checked with the respective species of FishBase and previous literature.

### 2.5 Estimation of Pedigree Index

It classifies the input sources of an Ecopath by analyzing the type of source data utilized and identifying the potential uncertainty linked to the input. The primary principles employed here are that input calculated from local data (i.e., from the area included by the model in question) is generally superior to data sourced from other locations, whether it be a guesstimate, obtained from different models, or based on empirical relationships. Three scales adhere to the aforementioned principles: the first for biomass assessment, the second for calculating P/B and Q/B ratios, and the third for diet composition, which varies between 0 and 1. A score near 0 signifies that the utilized input data is not grounded in local data, while a value approaching 1 shows that it is entirely anchored in local data. The fit measure (t*) is calculated to assess the model's alignment with local data.

### 2.6 Network Analysis and Ecosystem Indicators

Ecological network analysis demonstrates the quantitative and qualitative relationships, facilitating a deeper comprehension of the system's energy and nutritional transfer, as well as the conservation of circulating materials across the system (Scharler and Fath 2009). The “Mixed Trophic Impacts (MTI)” methodology, as described by Ulanowicz and Puccia (1990), was utilized in the EwE to assess the trophic effects of each functional group, incorporating fishing fleets, on every other group within an ecosystem. It involves both indirect and direct effects, including the interactions of predation and competition (Christensen et al. 2005). The MTI assesses the comparative effect of biomass changes of one functional group on other groups within the system. Predators adversely affect prey populations, while prey positively influence predator communities.

Ecosystem attributes in EwE were theoretically measured using the ecological theories proposed by Odum (1969) and the ecosystem's stability and maturity were assessed using a series of indicators (Christensen et al. 2005). Total system throughput (TST) is an important indicator that represents the overall size of flows within an ecosystem (Hossain et al. 2013). The connectance index (CI) and system omnivory index (SOI) were applied to ascertain whether the structure of the food chain is linear or web‐like. With the system maturation, trophic relationships switch from linear to a web‐like structure (Odum 1969). The CI represents the observed amount of food links within a system relative to the total possible links, which fluctuate according to system size and diet matrix (Hossain et al. 2013), whereas the SOI indicates the distribution of dietary interactions across trophic levels.

## 3 Results

### 3.1 Basic Analysis of the Model

**FIGURE 2:**
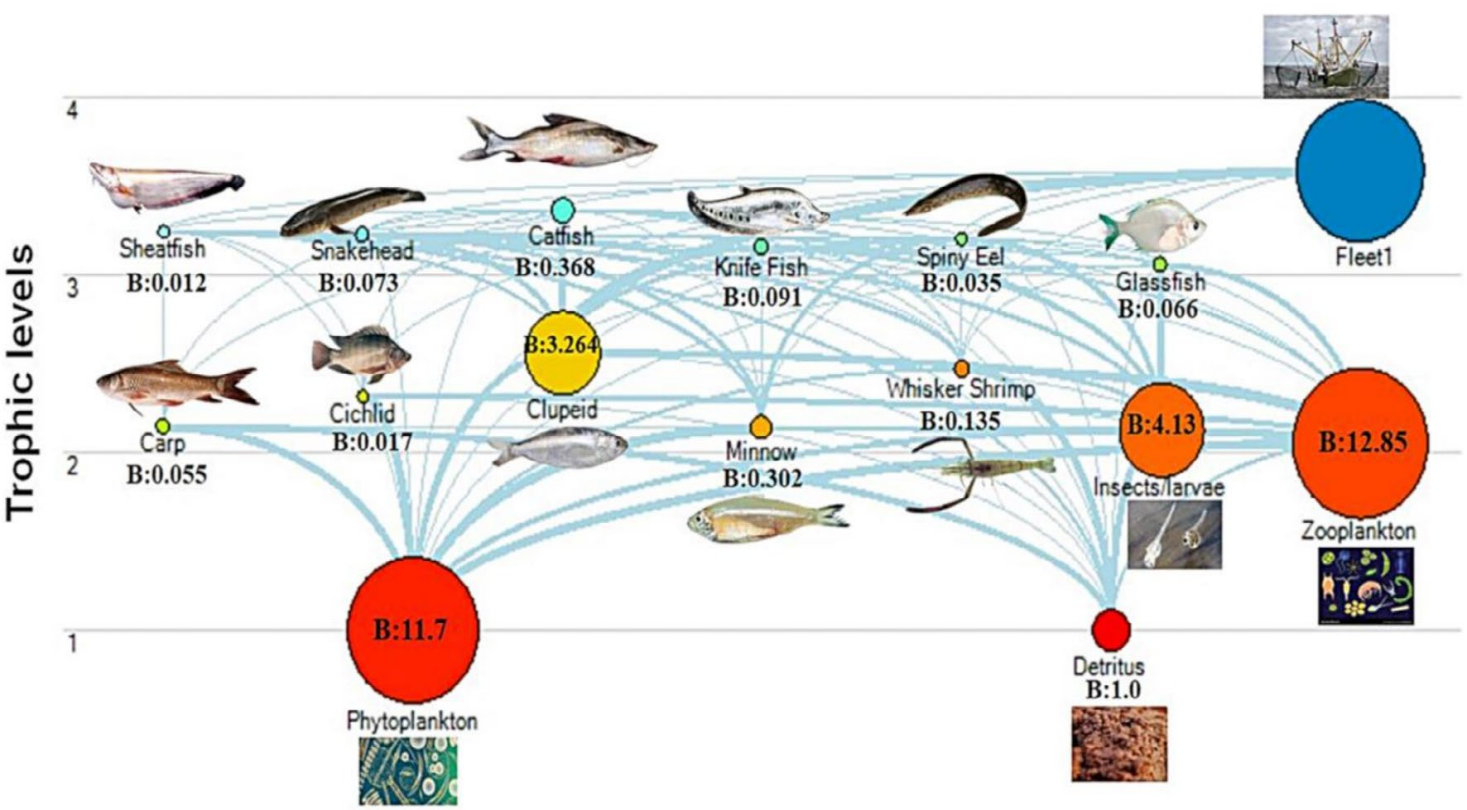
Diagram of energy flow illustrates the food web structure of the Kaptai Lake system. The gray lines indicate trophic levels 1, 2, and 3, while circle sizes reflect biomass (t/km^2^) of functional groups.

The model parameters and diet matrix have shown in Tables 2 and 3 respectively after calibration of the model. The model uncovered three trophic levels (TL) within the KL, with a mean TL of 2.58. Catfish (3.36) and Sheatfish (3.24) had the highest recorded values followed by Snakehead (3.22) and Spiny eel (3.20), while the predominant Clupeid‐TL was 2.56 (Figure 2; Table 2). The biomass of the fish groups in KL varied between 0.012 and 3.264 t/km^2^, with the highest value in Clupeid. The total fish biomass of KL was 4.420 t/km^2^. The EE values ranged from 0.228 to 0.998, with most of the functional groups showing EE values near to 1. Higher EE values were observed for Minnow, Whisker Shrimp, Carp, and Glassfish in this lake, whereas overabundant Clupeid (0.612) did not exhibit high EE and was at a lower level among fish groups, only higher than Sheatfish (0.551) and Knifefish (0.418). Additionally, Detritus showed the lowest values (0.228) among non‐fish groups (Table 2).

The ranges of values of P/Q, NE, and P/R of different groups were 0.043–0.257, 0.053–0.321, and 0.056–0.473, respectively, where the lowest and highest values of each parameter were in Minnow and Sheatfish, respectively. On the contrary, the lowest (0.679) and highest (0.946) values of R/A were in Sheatfish and Minnow, respectively. The highest (834.103) and lowest (0.041) values of FD were in Phytoplankton and Sheatfish, respectively, whereas the OI was highest (834.103) in Sheatfish, one of the apex predators, and the lowest values (0.041) were in Zooplankton (Table 2).

The diagnostic results of the model showed that the pedigree value was 0.662, with a measure of fit of 3.056 (Table 5), approaching the upper end of the range (0.16–0.68) of 150 Ecopath models (Morissette et al. 2006). This suggests that the parameter input values were derived from credible sources and the model can be considered acceptable with a high level of confidence (Christensen et al. 2005).

### 3.2 Ecosystem Structure and Trophic Analysis

**FIGURE 3:**
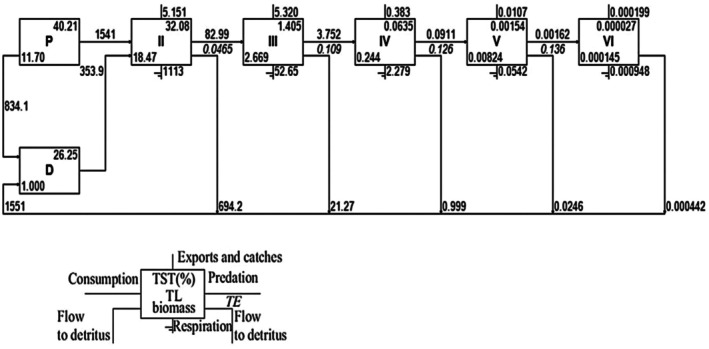
Lindeman spine of the Kaptai Lake ecosystem. D, Detritus; P, Primary producers; TE, Transfer efficiency; TL, Trophic level; TST, Total system throughput.

The KL food web structure showed relative biomass and energy transfers among functional groups at different trophic levels (Figure 2). Ecopath's network analysis simplifies the intricate food web, known as the “Lindeman spine,” which showed six distinct trophic levels (I to VI), encompassing all functional groups of the KL ecosystem (Lindeman 1942) (Figure 3). It estimates the energy flow and transfer efficiency across trophic levels. The Detritus food chain and primary producers contributed 35.12% and 64.88% in the KL food web respectively. The estimated total net primary production in KL was 2375.1 t/km^2^/year (Table 5). Total flows into the detritus pool were 2385.1 t/km^2^/year, and the consumed amount was 353.9 t/km^2^/year. The highest value of trophic flow (1894.9 t/km^2^/year) was from TL‐1 to TL‐2. The calculated flows from TL‐2 to TL‐3 and TL‐3 to TL‐4 were 82.99 t/km^2^/year and 3.752 t/km^2^/year, respectively (Figure 3).

The estimated mean transfer efficiency (TE) from TLII–TLIV was 8.629%, whereas primary producers and Detritus originated 8.446% and 9.352% of transfer efficiencies, respectively. The proportion of total flow originated from the Detritus was 0.33 (Table 4). The estimated highest flow to Detritus (FD) (834.103 t/km^2^/year) was contributed by the Phytoplankton in the system (Table 2).

**TABLE 4:** Transfer efficiency (%) at various trophic levels of the Kaptai Lake.

Source/Trophic level	II	III	IV	V	VI	VII	% values
Producer	4.405	10.87	12.58	13.55	14.01		8.446
Detritus	5.725	11.12	12.85	13.61			9.352
All flows	4.652	10.93	12.63	13.56	14.03	14.24	8.629

*Note:* Fraction of total flow originated by Detritus was 0.33.

### 3.3 Ecosystem Indicators

Table 5 has displayed all the ecological parameters of the KL ecosystem, as enumerated by the statistical routine of the EwE software and flow indicators (Christensen et al. 2005). The overall system throughput of the KL ecosystem was 5997.488 t/km^2^/year, with 34.54% arising from consumption (2071.815 t/km^2^/year), 20.13% from exports (1207.552 t/km^2^/year), 19.46% from respiration (1167.548 t/km^2^/year), and 25.85% (1550.573 t/km^2^/year) that ultimately ended up as Detritus. The total production (TP) of the system was 2865.004 t/km^2^/year whereas total net primary production (TNPP) and net system production (NSP) were 2375.1 t/km^2^/year and 1207.551 t/km^2^/year respectively. The ratios of total primary production to the total respiration (TPP/TR) and total biomass (TPP/TB) were 2.034 and 71.764 respectively. The mean trophic level of catch (MTLc) was estimated at 2.586, whereas the gross efficiency (catch/net primary production) was 0.0045 in the KL system. The computed total biomass/total system throughput (TB/TST) was 0.0055 t/km^2^/year, whereas total biomass (excluding Detritus) and total catch were 33.096 t/km^2^ and 10.865 t/km^2^/year respectively. Ecopath calculated the connectance index (CI), system omnivory index (SOI), Finn's cycling index (FCI) and Finn's mean path length (FMPL) at 0.426, 0.166, 5.627 (% of TST) and 2.525, respectively. The values of ascendancy (A) and system overhead (O) for KL were 32.09% and 67.91%, respectively, indicating the mean mutual information within the system (Table 5).

**TABLE 5:** Ecosystem features of Kaptai Lake.

Parameter	Value	Units	Parameter	Value	Units
Sum of all consumption	2071.815	t/km^2^/year	Total biomass (excluding Detritus)	33.096	t/km^2^
Sum of all exports	1207.552	t/km^2^/year	Total catch	10.865	t/km^2^/year
Sum of all respiratory flows	1167.548	t/km^2^/year	Connectance Index (CI)	0.426	—
Sum of all flows into Detritus	1550.573	t/km^2^/year	System Omnivory Index (SOI)	0.166	—
Total system throughput (TST)	5997.488	t/km^2^/year	Throughput cycled (including Detritus)	337.5	t/km^2^/year
Sum of all production (TP)	2865.004	t/km^2^/year	Ecopath pedigree	0.661	—
Mean trophic level of the catch (MTLc)	2.586	—	Finn's cycling index (FCI)	5.627	% of total throughput
Gross efficiency (catch/net p.p.) (GE)	0.0045	—	Finn's mean path length (FMPL)	2.525	—
Calculated total net primary production (TNPP)	2375.1	t/km^2^/year	Measure of fit, t*	3.056	—
Total primary production/total respiration (TPP/TR)	2.034	—	Ascendency (A)	32.09	—
Net system production (NSP)	1207.551	t/km^2^/year	Overhead	67.91	t/km^2^/year
Total primary production/total biomass (TPP/TB)	71.764	—	Loss in production index (L index)	0.113	—
Total biomass/total system throughput (TB/TST)	0.0055	t/km^2^/year	Shannon diversity index	1.405	—

### 3.4 MTI Routine

**FIGURE 4:**
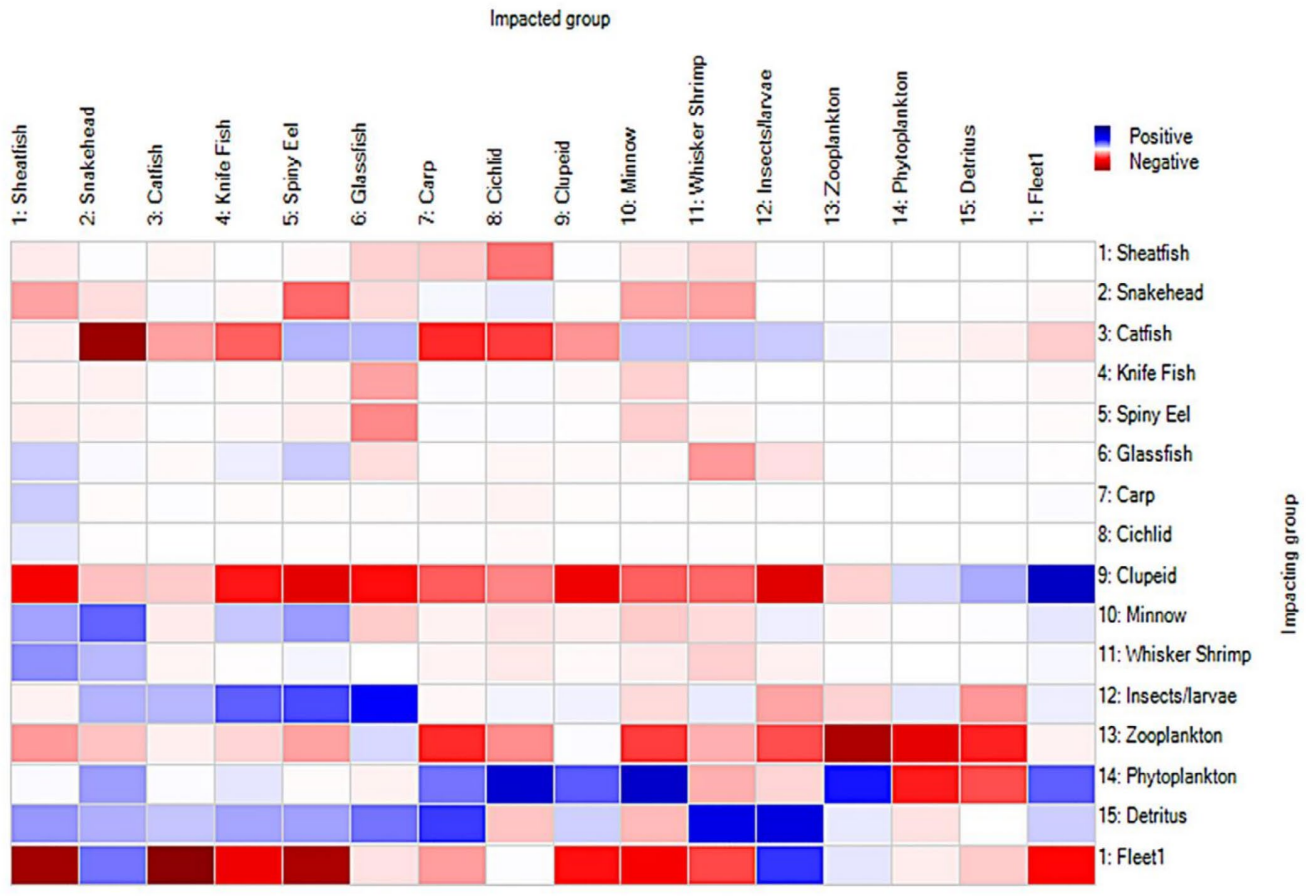
Mixed trophic impact (MTI) study of Kaptai Lake system. The blue bars indicate positive impact, whereas red bars represent negative impact. The intensity of the colors signifies the magnitude of impact.

In the KL ecosystem, Detritus and Phytoplankton positively impacted the majority of the functional groups, particularly their direct predators, emphasizing their significance in the ecosystem via bottom‐up regulation. Clupeids negatively impacted the majority of the other fish groups, whereas Catfish positively impacted Spiny eel, Glassfish, Minnow, Whisker shrimp, and Insects/larvae while negatively impacting Snakehead, Knifefish, Carp, and Cichlid. Most of the fish groups in the same trophic level showed minor impacts on themselves, either positively or negatively. On the contrary, except for Detritus, every functional group of lower trophic levels is negatively impacted by themselves, indicating competition for common food sources (Christensen et al. 2005) (Figure 4).

### 3.5 Keystone Index

**FIGURE 5:**
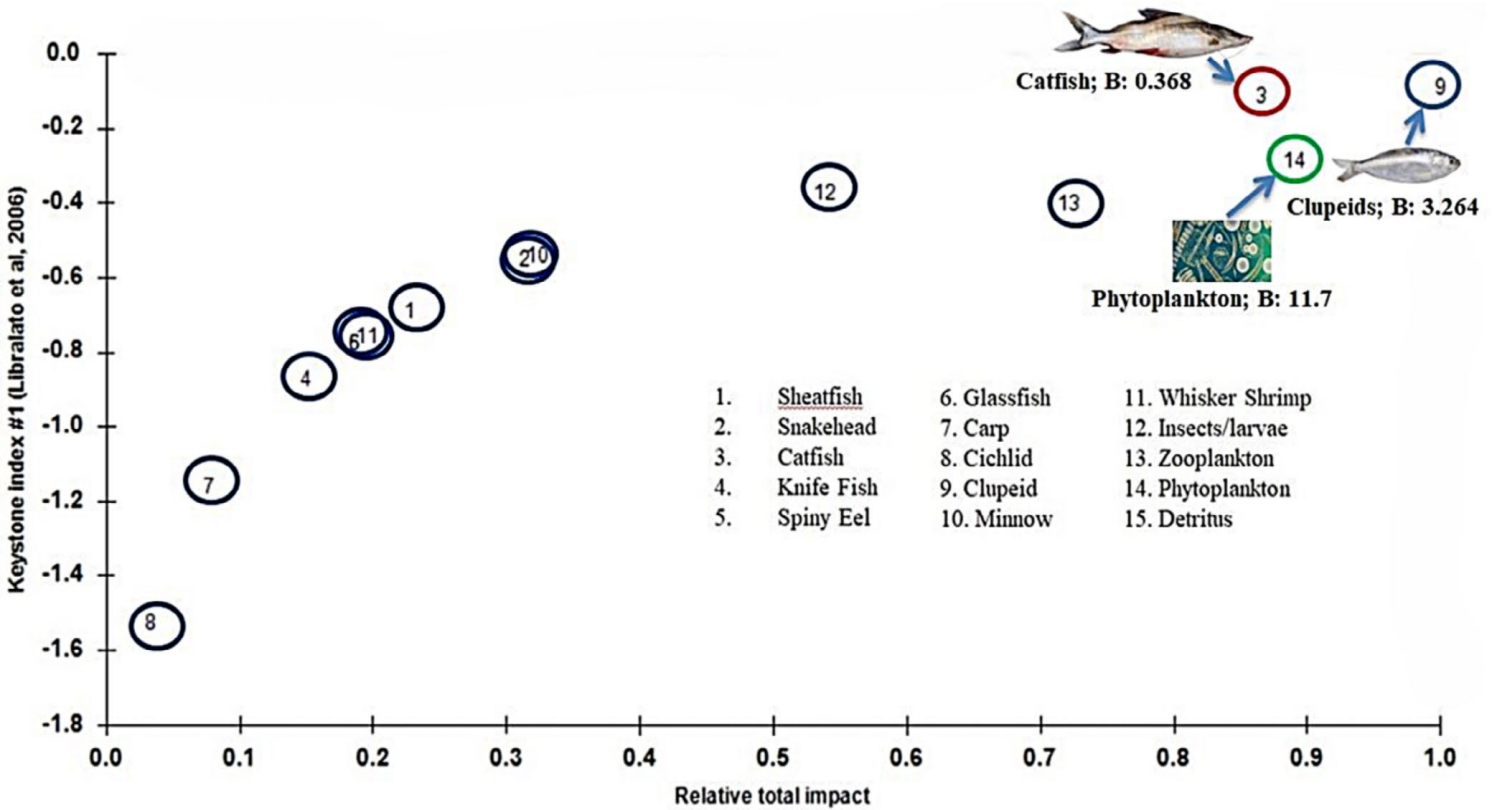
Keystone index of different functional groups of Kaptai Lake system.

Among the predator groups, Clupeid had the highest relative total impact (1.0), followed by Catfish (0.867) and thereby these two groups were considered the key predator group in the KL ecosystem while the key producer group was Phytoplankton with the highest relative total impact of 0.891 (Figure 5).

### 3.6 Niche Overlap

**FIGURE 6:**
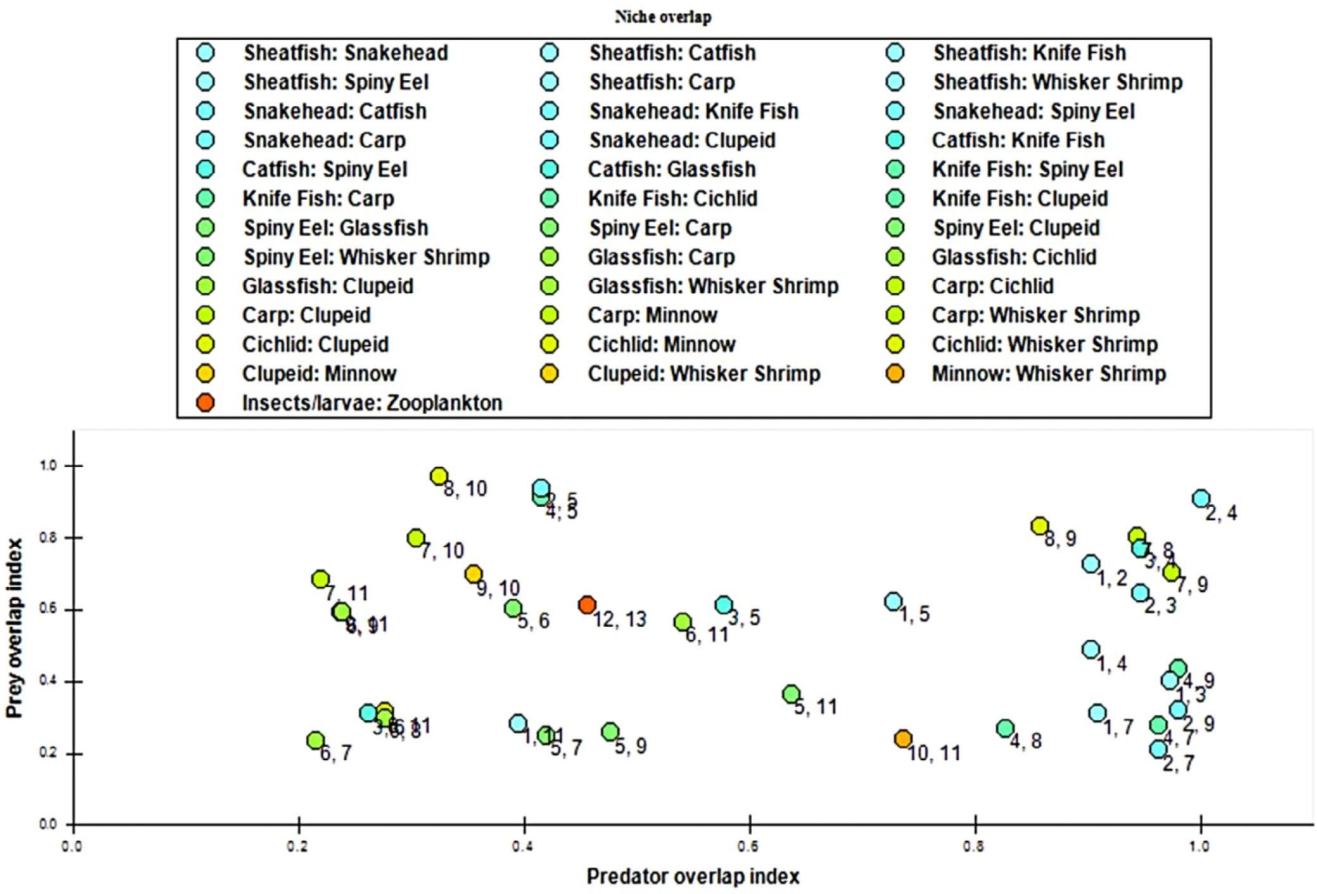
The niche overlap plot of Kaptai Lake system. The threshold for the predator and prey overlap indicator scale has been determined at 0.2.

Numerous overlap indices have been proposed over the years to quantify species interspecific overlap. Pianka (1973) suggests that number ‘0’ indicates no resource sharing between the two species, whereas number ‘1’ implies absolute overlap and intermediate values indicate a partial overlap for utilization of resources. The niche overlap plot (Figure 6) in the current study shows that the maximum prey overlap was observed between Chiclid and Minnow (group 8 and group 10) followed by Snakehead–Spiny eel (group 2 and 5) and Knifefish–Spiny eel (group 4 and 5). The greatest niche overlap was observed between Snakehead and Knifefish (group 2 and 4) in terms of their predators and preys. The highest predatory overlap was also noticed between Snakehead and Knifefish suggesting intense competition for food and habitat (Pianka 1973).

## 4 Discussion

### 4.1 Food Web Pattern of Kaptai Lake

**FIGURE 7:**
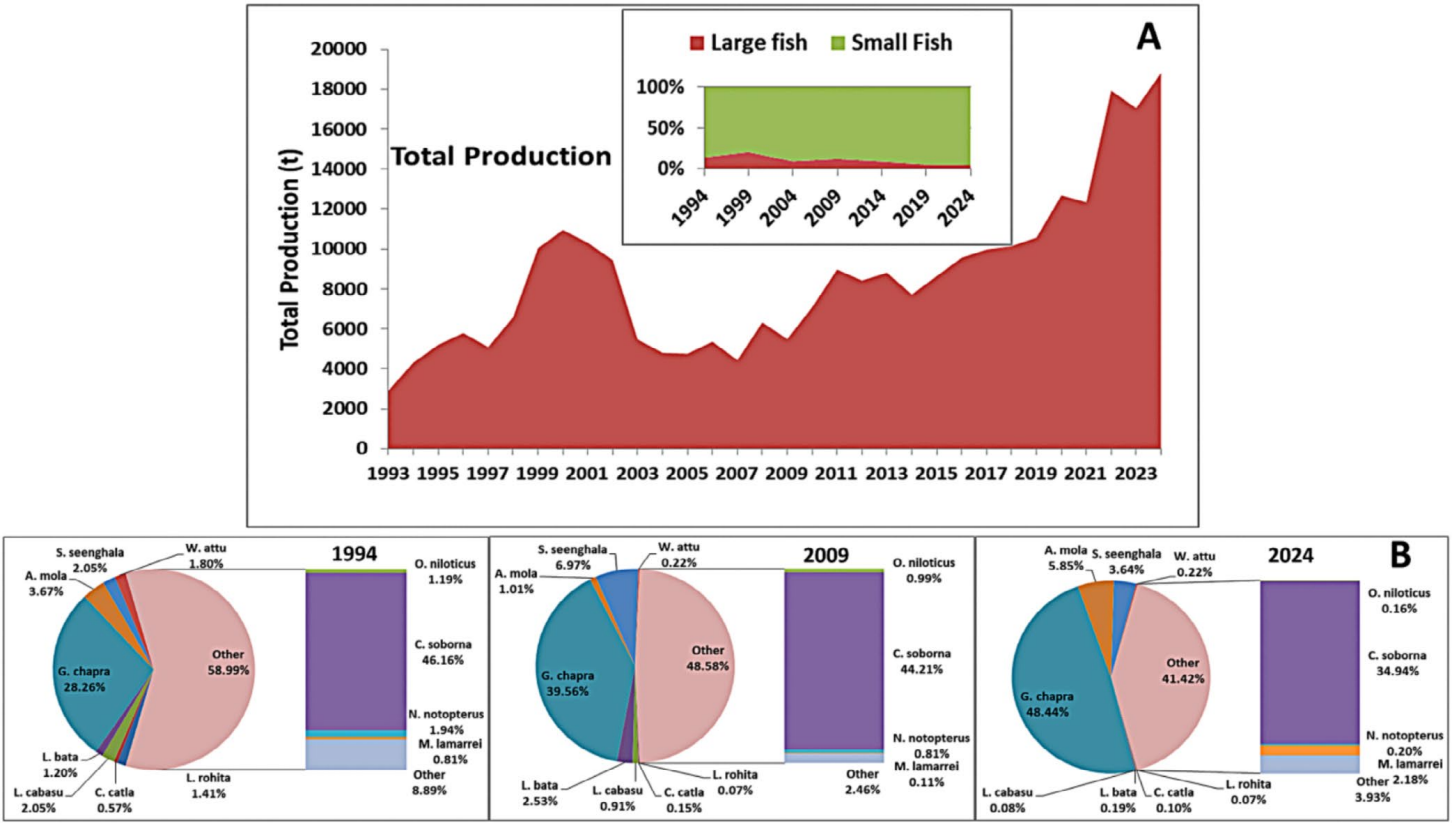
(A) Total landings (t) from 1993 to 2023 and the proportion of large and small‐sized fish in the total production. (B) Catch composition of the total production in 1994, 2009, and 2024. Full name of all species has been shown in Table 1.

**FIGURE 8:**
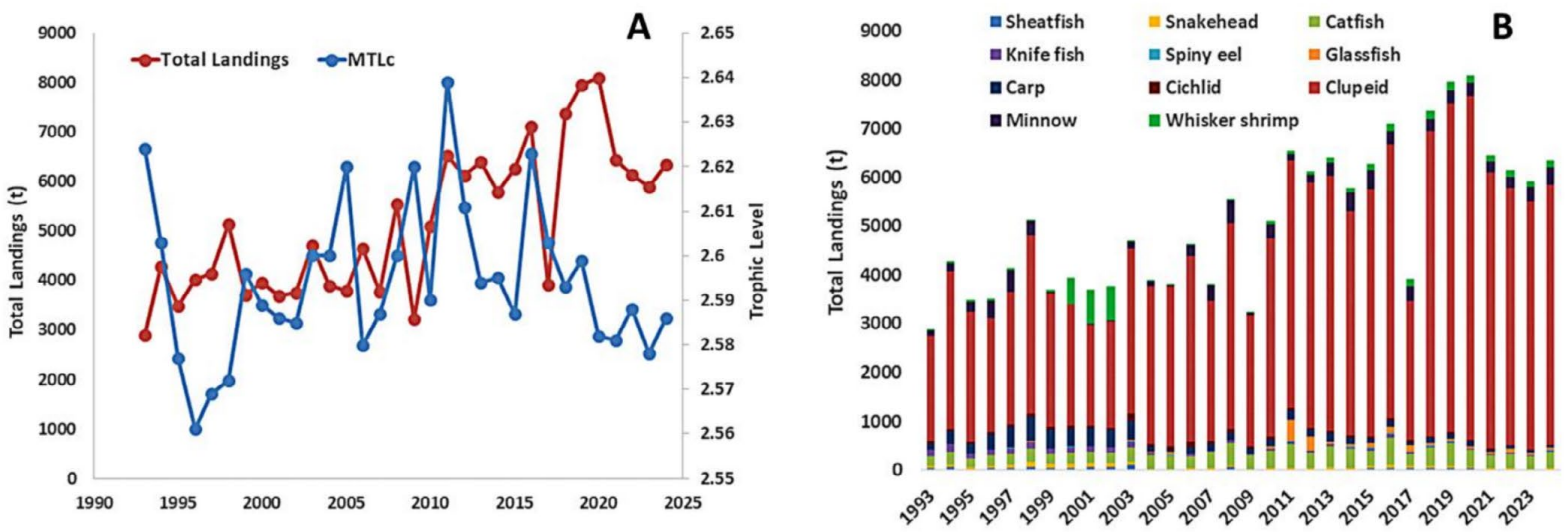
(A) Mean trophic level of total landings and (B) group‐wise total landings of major commercial species in the Kaptai Lake from 1993 to 2024.

Ecological assessment in Ecopath reveals the overall scenario of an ecosystem and represents the current resources together with their interactions via food consumption. KL had a much lower total fish biomass than Bakreswar reservoir (53.00 t/km^2^) and Lake Ayame (8.02 t/km^2^) but higher than that of Great Bear Lake (0.89 t/km^2^) (Banerjee et al. 2016; Traore et al. 2008; Janjua et al. 2015). Among the fish groups, Sheatfish and Knifefish had the lowest value of EE, which was reasonable and aligns with expectations for the higher‐level predators (Christensen et al. 2000), but the high value of EE (> 0.7) in the Carp (0.971), Snakehead (0.95), and Catfish (0.701) indicates heavy fishing pressure on these targeted commercially important groups by the multispecies gear. For Clupeid, which currently has the highest abundance in the lake, the EE values (0.612) indicate that the fish is not well utilized, which might be due to the decrease of their major predators especially Sheatfish, Knifefish, Snakehead, and Catfish (Figures 7 and 8B). Besides, fishing pressure on Clupeid is not sufficient compared to their production. A comparably lower value of EE for Zooplankton (0.353) than Phytoplankton (0.649) is possibly due to an insufficient number of zoophagous fish groups in the system. The lowest EE value (0.228) of Detritus indicates that it is not a highly exploited resource, whereas a significantly higher value of Phytoplankton indicates their availability in the diets of fish groups with lower trophic levels. This finding agrees with the previous study of Lake Ziway, Bakreswar reservoir, Hongze Lake, and Tonle Sap Lake (Hailu et al. 2022; Banerjee et al. 2016; Guo et al. 2018; Chea et al. 2016). The relatively higher EE value of primary producers demonstrates the ability of ecosystem for bottom‐up regulation (Traore et al. 2008).

The food chain of the KL ecosystem is Phytoplankton‐based, where Phytoplankton and Detritus make up the majority of the KL food web. Therefore, the prevalence of primary producers relative to primary consumers generates the prey necessary for sustaining the highest trophic levels, thereby supporting lake fisheries (Pauly and Christensen 1995). Higher transfer efficiency of flows from Detritus compared to Phytoplankton indicates the crucial role of the Detritus in the food chain of this system (Table 4). The mean transfer efficiency of all flows in KL was higher than Lake Ziway (4.4%) and Lake Great Bear (8.1%), while it was lesser than Lake Kawahara‐oike (14.9%) and Lake Victoria (10.55%) (Hailu et al. 2022; Janjua et al. 2015; Hossain et al. 2013; Natugonza et al. 2020). The comparatively higher TE of KL supports diversified predators of the system. The value of TE was gradually increased from TL II to TL VII, which is supported by Lindeman's theory (Lindeman 1942).

The MTI study demonstrated that the majority of the other groups were positively impacted by Phytoplankton and Detritus, especially bottom dwellers, i.e., Whisker Shrimp, Insects/larvae, and Carp, indicating a bottom‐up effect from an ecosystem perspective (Khatun et al. 2021; Dutta et al. 2023). There was a noticeable negative impact of Zooplankton on itself, which suggests that a large amount of carnivorous Zooplankton is present in the system (Figure 7). Their negative impact on Phytoplankton indicated that there was a smaller fraction of herbivorous Zooplankton in the system. Most of the groups showed minor negative impact or no impact on Clupeid, indicating a lack of major predators of this group, which could potentially contribute to the dramatic increase of Clupeid population in the lake. The predators of Clupeid especially *Wallago attu* , *Notopterus notopterus* and *Sperata seenghala* has decreased over the periods (Figure 7). Suman et al. (2021) and Khatun et al. (2021) also showed the declining trend of predators of Clupeid like *Wallago attu* , *Notopterus chitala* and *Channa marulius* in their studies. The positive impacts of Catfish on most of the fish groups further validated its carnivorous nature as a top predator. The negative effect of the Fleet‐One upon majority groups implies considerable fishing pressure. Consequently, further fishing may adversely affect higher predators.

In the KL ecosystem, the higher relative total impact of the keystone groups (Catfish, Clupeid, and Phytoplankton) indicates their significance in the system. Keystone species are extremely important in terms of climate change. A considerable reaction of keystone species to climate change could profoundly affect food web structure (Blenckner 2005), particularly in simple food webs like the KL ecosystem.

### 4.2 Ecosystem Indicators and Properties

Some key ecological indicators in Ecopath seem to effectively measure the level of maturity, balance, and overall health of an ecosystem (Christensen 1995). The TST of KL was 5997.488 t/km^2^/year which was consistent with the findings of Lake Malawi (6184 t/km^2^/year) and Bakreswar reservoir (4435.965 t/km^2^/year) (Darwall et al. 2010; Banerjee et al. 2016). The estimated lower value of the MTLc (2.586) indicates the lack of specific apex predators and the comparatively short food chain of the system. It also indicates that the top and mid‐level predators are the extremely targeted groups for fishing in this system. After 1990, KL experienced a sharp fall of MTLc due to the overexploitation of Carps and pronounced proliferation of Clupeid and Minnow. Subsequently, some ranching and enhancement initiatives for carp fingerlings led to a modest rebound in MTLc in the following years; however, over the past decade, MTLc has again exhibited a gradual decline due to the massive contribution of low trophic level Clupeid group in the total landings (Figure 8). Previous research has shown that overfishing can have a major impact on a lake's trophic structure, resulting in a shift in the regime from big and slow‐growing k‐selected species to smaller but more rapidly reproducing r‐selected species (Travers et al. 2010). A reduction of bigger fish from the upper trophic levels can result in an increase in smaller fish, demonstrating the idea of “fishing down the food web” (Pauly et al. 1998). This can be evidenced in the KL by the higher EE values of most of the top predators due to heavy fishing pressure, the massive biomass of small fish like Clupeids (3.264 t/km^2^) compared to large fishes like Carp (0.055 t/km^2^), Sheatfish (0.012 t/km^2^), and Catfish (0.368 t/km^2^) (Table 2) and the opposite trend of historical landings of small and large fishes (Figure 7). Several studies on this lake have already proved the overexploitation of different high‐valued fish species, especially Carps, and showed increasing concern for the dominance of small fish like Clupeids in this lake (Khatun et al. 2023; Rahman et al. 2024; Suman et al. 2021). If substantial fishing pressure continues on most of the apex predators, the KL ecosystem will face disastrous consequences such as food web collapse and catastrophic regime shifts in the near future.

The TPP/TR ratio and TPP/TB ratio are two critical indices for assessing ecosystem maturity (Odum 1969). Matured ecosystems often exhibit a TPP/TR ratio approaching 1 and a lower value of TPP/TB. The TPP/TR ratio (2.034) of KL indicates a developing ecosystem with a certain level of system maturity, which was consistent with the findings of Lake Ziway (2.18) and Bakreswar Reservoir (1.721) (Table 6) (Hailu et al. 2022; Banerjee et al. 2016). The estimated TPP/TB ratio (71.764) of KL reconfirmed the ecosystem's growing feature that has already been addressed. Similar finding was observed for Ravishankar Sagar reservoir (80.33) and Lake Malawi (66.0) but it was much higher than Tonle Sap Lake (2.039) and Lake Great Bear (4.79) (Panikkar et al. 2015; Darwall et al. 2010; Chea et al. 2016; Janjua et al. 2015). The cycling of both energy and matter is regarded as a crucial mechanism in the operation of any ecosystem (Odum 1969). FCI corresponds with the system's overall steadiness, resiliency, and maturity (Finn 1976). A higher FCI value indicates a more stable and matured ecosystem (Odum 1969). The study of FCI for KL showed that the value was 5.627%, which is quite low when compared with Lake Gehu (14.76%) and Tonle Sap Lake (23.62%) but slightly higher than Lake Erhai (1.98) and Lake Koka (2.23) (Jia et al. 2012; Chea et al. 2016; Yin et al. 2022; Tesfaye and Wolff 2018).

**TABLE 6:** Comparative scenario of Kaptai Lake Ecopath model with other similar ecosystems.

Ecosystem	TST	GE	TPP/TR	TPP/TB	TB/TST	NSP	MTLc	CI	SOI	A	FCI	FMPL
Kaptai^a^	5997.488	0.0045	2.034	71.764	0.005	1207.55	2.586	0.426	0.166	32.09	5.627	2.525
Bakreswar^b^	4435.965	—	1.721	9.733	0.05	908.223	—	0.176	0.11	—	—	—
Tonle Sap^c^	14050.33	0.007	1.23	2.039	0.122	654.194	2.482	0.253	0.075	0.274	23.62	4.015
Great Bear^d^	118.86	0.00008	1.44	4.79	0.07	12.25	3.55	0.29	0.09	33.02	10.58	2.96
Erhai^e^	20,243.28	—	5.70	11.37	0.04	7287.41	2.62	0.21	0.16	38.86	1.98	2.29
Koka^f^	9355.489	0.08	3.198	16.157	0.027	—	2.55	0.405	0.289	11313.6	2.23	—
Chaohu^g^	24541.85	0.002	2.132	111.15	0.004	5676.64	2.912	0.238	0.075	38.6	0.09	2.261
Victoria^h^	9305.8	—	1.33	25.3	0.012	742.7	—	0.32	0.12	12,110	4.75	3.12
Ziway^i^	10984.4	0.00056	2.18	19.406	—	2328.97	2.49	0.306	0.146	—	—	—
Hongze^j^	23,141.94	0.017	1.138	6.922	0.051	984.556	2.094	0.195	0.089	0.216	6.77	2.849

^a^ Present study.

^b^ Banerjee et al. (2016).

^c^ Chea et al. (2016).

^d^ Janjua et al. (2015).

^e^ Yin et al. (2022).

^f^ Tesfaye and Wolff (2018).

^g^ Kong et al. (2016).

^h^ Natugonza et al. (2020).

^i^ Hailu et al. (2022).

^j^ Guo et al. (2018).

Ascendancy and overhead are two other indexes that also correlate with the stability, maturity and overall health of the ecosystem (Christensen 1995). The lower value of ascendancy (32.09%) compared to Ravishankar Sagar Reservoir (46.8%) and Lake Koka (11313.6%), along with a higher overhead value (67.91%) of KL in contrast to Great Bear Lake (47.3%) and Lake Chaohu (61.4%), indicated a maturing ecosystem with strong stability (Panikkar et al. 2015; Tesfaye and Wolff 2018; Janjua et al. 2015; Kong et al. 2016). The moderately lower connectance index (CI: 0.426) and higher omnivory index (0.166) of KL indicate that the food web is still linear, rather than a web‐like structure with some degree of system maturity as well and diversified diets of certain functional groups in the system (Christensen et al. 2005).

### 4.3 Ecosystem‐Based Management Approaches for Restoration of Kaptai Lake

It has been widely accepted that the ecosystem‐based fisheries management approach is crucial for managing sustainable fisheries and ecosystem health. Our current Ecopath model suggests that the KL ecosystem remains in a developmental stage and is highly exploited, especially by top predators. At the same time, it is extremely susceptible to perturbations, as it is a developing ecosystem with simpler trophic interactions that could be greatly impacted by prospective threats.

Since the last decade, the biggest concern of the lake is the gradual decline of large‐sized fish, especially Carps and apex predators, and the dramatic overgrowth of small fish, including Clupeids (Rahman et al. 2024; Khatun et al. 2023; Suman et al. 2021). Therefore, based on the present study, our first and foremost suggestion is to stop the overexploitation of all types of vulnerable species immediately, especially Carps, Sheatfish, Knifefish, and Snakehead. Besides, it is also quite urgent to restore the destroyed natural breeding grounds of Carps by excavating the connecting channels of the lake and river. Apart from this, it is necessary to control the illegal fishing of Carp fingerlings during the catching of small fish using small meshed seine nets (mosquito nets). We are also suggesting the ranching and enhancement of native fishes, especially carnivore and omnivore species, and the stocking of more Carp fingerlings in the lake, as numerous studies already showed that it helps to increase species richness and strengthen trophic structure and ecosystem function (Guo et al. 2018). Moreover, the carnivorous fish can significantly reduce the overgrowth of small fish by predation and also help to decrease the ever‐increasing competition of Carp fingerlings with small fishes for food and space. However, we also suggest building a dynamic simulation model (Ecosim) based on our existing Ecopath model to forecast possible consequences of fish stocking in the lake. Invading species are globally known to modify community composition, energy dynamics, food web pattern, and ecosystem attributes (Guo et al. 2018). In Kaptai Lake, tilapia is the major invasive species with greater population size (DoF 2023). Therefore, the ecological effects of invasive species need to be assessed for better understanding of ecosystem health. Finally, it is imperative to implement an integrated and long‐term policy for surveillance networks to deliver comprehensive first‐hand data, which can assist scientists and ecosystem managers in making more sensible decisions for the sustainable management of this lake.

## Author Contributions

**Debashis Kumar Mondal:** conceptualization (supporting), data curation (lead), formal analysis (lead), software (lead), writing – original draft (lead). **Chengjie Yin:** writing – review and editing (lead). **Kangshun Zhao:** conceptualization (supporting), supervision (supporting), writing – review and editing (supporting). **Muhammad Farooq:** writing – review and editing (supporting). **Md. Abdul Halim:** writing – review and editing (supporting). **Ruilong Wang:** software (supporting), writing – review and editing (supporting). **Jun Xu:** conceptualization (lead), funding acquisition (lead), supervision (lead), writing – review and editing (supporting).

## Funding

This work was supported by the Chinese Scholarship Council (CSC).

## Conflicts of Interest

The authors declare no conflicts of interest.

## Supporting information


**Table S1:** Source of data of biomass, population parameters and diet of different functional groups in Kaptai Lake.

## Data Availability

The data that support the findings of this study are available in figshare at: https://doi.org/10.6084/m9.figshare.31150237.
