# Supplementary material for: Modeling Trophic Structure and Ecosystem Functioning of the Small Fish‐Dominated Largest Lake of Bangladesh
Source: Ecol Evol. 2026 Mar 14;16(3):e73177. doi: 10.1002/ece3.73177 (PMC13093402; doi:10.1002/ece3.73177)
Supplement: Supplementary file 1 — Table S1: Source of data of biomass, population parameters and diet of different functional groups in Kaptai Lake. [file ECE3-16-e73177-s001.docx]

Table S1: Source of data of biomass, population parameters and diet of different functional groups in Kaptai Lake.

| **Functional groups** | **Biomass** | **L∞ (cm)** | **K (/yr)** | **a** | **b** | **W∞**  **(g)** | **M (/yr)** | **F (/yr)** | **Z (/yr)** | **E** | **Diet** | **Reference for diet** |
| --- | --- | --- | --- | --- | --- | --- | --- | --- | --- | --- | --- | --- |
| Sheatfish | LD | SS | SS | SS | SS | EM | SS | SS | SS | SS | SS | Babare et al., 2013; Gupta, 2018 |
| Snakehead | LD | LD | LD | LD | LD | EM | LD | LD | LD | LD | SS | Rao et al., 1998; Bhuiyan et al., 2006 |
| Catfish | LD | LD | LD | LD | LD | EM | LD | LD | LD | LD | LD | Azadi et al., 1990; Azadi et al., 1991 |
| Knifefish | LD | LD | LD | LD | LD | EM | LD | LD | LD | LD | LD | Azadi et al., 1994 |
| Spiny eel | LD | SS | SS | SS | SS | EM | SS | SS | SS | SS | SS | Kashyap et al., 2023 |
| Glassfish | LD | SS | SS | SS | SS | EM | SS | SS | SS | SS | SS | Bhuvaneswari and Serfoji, 2018 |
| Carp | LD | LD | LD | LD | LD | EM | LD | LD | LD | LD | LD | Azadi and Naser, 1996; Imran et al., 2014 |
| Cichlid | LD | LD | LD | LD | LD | EM | LD | LD | LD | LD | LD | Azadi and Nasiruddin, 1990 |
| Clupeid | LD | LD + FishBase | LD + FishBase | LD + FishBase | LD + FishBase | EM | LD + FishBase | LD + FishBase | LD + FishBase | LD + FishBase | LD | Rahman et al., 2008 |
| Minnow | LD | LD | LD | LD | LD | EM | LD | LD | LD | LD | LD | Mamun et al., 2004 |
| Whisker Shrimp | LD |  |  |  |  |  |  |  | SaS |  | SaS | Khatun et al., 2021 |
| Insects/larvae | SaS |  |  |  |  |  |  |  |  |  | SaS | Khatun et al., 2021 |
| Zooplankton | SaS |  |  |  |  |  |  |  |  |  | FishBase | Froese and Pauly, 2025 |
| Phytoplankton | SaS |  |  |  |  |  |  |  |  |  |  |  |
| Detritus | SaS |  |  |  |  |  |  |  |  |  |  |  |

Note: LD = Local data, SaS = Same system, SS = Similar system, EM = Estimated in this model.

**References**

Azadi, M.A. and Naser, A. (1996). Food and feeding habits of *Labeo bata* (Hamilton) (Cyprinidae: Cypriniformes) from Kaptai reservoir’, *Chittagong University Studies, Part-II: Science*, 20 (2), pp. 11-17.

Azadi, M.A., and Nasiruddin, M. (2020). Some aspects of biology of *Oreochromis niloticus* (Linn.) from Kaptai lake,’ *Bangladesh Journal of Scientific Research (Special Issue)*, pp. 59-68.

Azadi, M.A., Islam M.A. and Solaiman, S. (1991). Food and feeding habits of *Eutropiichthys vacha* (ham.) from the Kaptai reservoir’, *Journal of the Asiatic Society of Bangladesh, Science*, 17 (1), pp. 53-60.

Azadi, M.A., Islam, M.A. and Paul, J.G. (1990, October). Biology and fishery of the catfish, Mystus aor, in the Kaptai reservoir, Bangladesh. In Sena S. De Silva (Eds.), *Proceedings of the 2nd Asian reservoir fisheries workshop held in Hangzhou, People's Republic of China* (pp. 125-140).

Azadi, M.A., Islam, M.A., Nasiruddin, M. and Quader, M.F. (1994). Food and feeding habits of a feather back, *Notopterus notopterus* (pallas) (Notopteridae: Clupeiformes) from the Kaptai reservoir,’ *Chittagong University Studies, Part-II: Science*, 18 (2), pp. 183-190.

Babare, R.S., Chavan, S.P. and Kannewad, P.M. (2013). Gut Content Analysis of *Wallago attu* and *Mystus (Sperata) seenghala* The Common Catfishes from Godavari River System in Maharastra State,’ *Advances in Bioresearch*, 4(2), pp. 123–128.

Bhuiyan, A.S., Afroz, S., and Zaman, T. (2006). Food and feeding habit of the juvenile and adult snakehead, *Channa punctatus* (Bloch),’ *Journal of Earth and Life Science*, 1(2), pp. 53–54.

Bhuvaneswari, R., and Serfoji, P. (2018). Studies on Growth and Feeding Biology of *Chanda Nama* (Hamilton, 1822 ) from Vettar River of Cauvery River Basin Nagore, Tamil Nadu,’ *International Journal of Innovative Research In Technology*, 175 4(8), pp. 175–182.

Gupta, S. (2018). A Review on Feeding and Reproductive Biology of Ompok pabda with an Emphasis on its Conservation,’ *Journal of Aquaculture Research and Development*, 9(2), pp. 525. https://doi.org/10.4172/2155-9546.1000525

Imran, S., Nagar, S., & Jha, D. N. (2014). A review: food and feeding habit of *Labeo calbasu* (Hamilton, 1822) from different habitat,’ *Journal of the Kalash Science*, 2(1), pp.71–73.

Kashyap, D. K., Mogalekar, H.S., Sahil, S.K., Nayak, R.K., Ram, Sharma, A., Kumar, P., Saxena M., Kumar, S. & Singh, M. K. (2023). Feeding habits and reproductive behavior of Zig-Zag Eel *Mastacembelus armatus* (Lacepède 1800) from the Burhi Gandak river in Bihar, India,’ *Journal of Inland Fisheries Society of India*, 55(1), pp. 03-15. https://doi.org/10.47780/jifsi.55.1.2023.141451

Khatun, M.H., Barman, P.P., Yi, J., Lupa, S.T., Zahangir, M.M. and Liu, Q. (2021) ‘A preliminary snapshot of the trophic model and ecosystem attributes of Kaptai reservoir ecosystem, Bangladesh’, *Journal of Oceanology and Limnology*, 39(1), pp. 223–241. <https://doi.org/10.1007/s00343-020-9284-5>

Mamun, A., Tareq, K.M.A. and Azadi, M.A. (2004). Food and feeding habits of *Amblypharyngodon mola* (Hamilton) from Kaptai reservoir, Bangladesh,’ *Pakistan Journal of Biological Sciences*, 7(4), pp. 584-588.

Rahman, M.A., Haque, M.M. and Khan, S. (2008). Food and feeding habits of chapila, *Gudusia chapra* (Hamilton-Buchanan) from Rajdhala reservoir,’ *Journal of Inland Fisheries Society of India*, 40 (2), pp. 13-20.

Rao, L.M., Ramaneswari, K., and Rao, L.V. (1998). Food and feeding habits of Channa species from East Godavari District (Andhra Pradesh),’ *Indian Journal of Fisheries*, 45(3), pp. 349–353.
